# Nothing is safe: Intolerance of uncertainty is associated with compromised fear extinction learning

**DOI:** 10.1016/j.biopsycho.2016.05.001

**Published:** 2016-12

**Authors:** Jayne Morriss, Anastasia Christakou, Carien M. van Reekum

**Affiliations:** Centre for Integrative Neuroscience and Neurodynamics, School of Psychology and Clinical Language Sciences, University of Reading, Reading, UK

**Keywords:** Emotion, Anxiety, Fear extinction, Intolerance of uncertainty, Skin conductance

## Abstract

Extinction-resistant fear is considered to be a central feature of pathological anxiety. Here we sought to determine if individual differences in Intolerance of Uncertainty (IU), a potential risk factor for anxiety disorders, underlies compromised fear extinction. We tested this hypothesis by recording electrodermal activity in 38 healthy participants during fear acquisition and extinction. We assessed the temporality of fear extinction, by examining early and late extinction learning. During early extinction, low IU was associated with larger skin conductance responses to learned threat vs. safety cues, whereas high IU was associated with skin conductance responding to both threat and safety cues, but no cue discrimination. During late extinction, low IU showed no difference in skin conductance between learned threat and safety cues, whilst high IU predicted continued fear expression to learned threat, indexed by larger skin conductance to threat vs. safety cues. These findings suggest a critical role of uncertainty-based mechanisms in the maintenance of learned fear.

## Highlights

• We tested how fear extinction learning varied with Intolerance of Uncertainty (IU).
• High IU predicted threat generalization during early extinction.
• High IU predicted continued responding to learned threat during late extinction.
• Future threat uncertainty may maintain learned fear in the anxiety disorders.

## 1 Introduction

The ability to discriminate between threat and safety is crucial for survival. Through fear conditioning, an organism can associate neutral cues (conditioned stimulus, e.g. a visual stimulus such as a shape) with aversive outcomes (unconditioned stimulus, e.g. shock, loud tone). Repeated presentations of a neutral cue with an aversive outcome can result in fearful responding to the neutral cue alone (conditioned response). This learned association can also be extinguished by repeatedly presenting the learned threat cue without the aversive outcome, a process known as fear extinction (LaBar, Gatenby, Gore, LeDoux, & Phelps, 1998; Milad & Quirk, 2002; Phelps, Delgado, Nearing, & LeDoux, 2004). During fear extinction, a reduction in reactivity to the learned threat cue over time is thought to reflect changes in harm expectancy and contingency beliefs (for a review see, (Hofmann, 2008)). Such fear extinction processes, however, are thought to be disrupted by cognitive biases – including attentional and expectancy biases – in individuals with anxiety and trauma disorders (Aue & Okon-Singer, 2015), who display delayed fear extinction or even extinction-resistant fear (Graham and Milad, 2011, Milad and Quirk, 2012, Mineka and Oehlberg, 2008). For example, compared to healthy controls, patients show elevated autonomic nervous system activity to both learned threat and safety cues at the start of extinction, and to learned threat cues across fear extinction learning (Blechert, Michael, Vriends, Margraf, & Wilhelm, 2007; Michael, Blechert, Vriends, Margraf, & Wilhelm, 2007; Milad et al., 2008, Milad et al., 2009).

In addition to examining fear extinction processes in clinical samples, it is important to test individual differences in non-clinical samples, to appropriately separate those processes that are risk factors for anxiety disorder development from those processes that are consequential to an anxiety disorder (Chambers, Power, & Durham, 2004). In two recent meta-analyses, however, only small differences in fear extinction behavior were found between anxious and non-anxious individuals (Duits et al., 2015, Lissek et al., 2005). Furthermore, findings have also been mixed from studies examining fear extinction behavior and trait anxiety, as measured with the Spielberger State-Trait Anxiety Inventory (STAI; Spielberger, Gorsuch, Lushene, Vagg, & Jacobs, 1983). For example, trait anxious individuals have been shown to display slower reductions in startle reactivity to both threat and safety cues during extinction (Gazendam, Kamphuis, & Kindt, 2013), but not in skin conductance (Haaker et al., 2015) or expectancy ratings (Barrett and Armony, 2009, Gazendam et al., 2013). These equivocal findings may stem from a lack of alignment between the STAI measure and the underlying cognitive mechanisms that disrupt fear extinction. For example, items in the STAI broadly address physical fear and anxiety symptoms or worrying, but items in the STAI do not capture any specific elicitors of fear and anxiety that may be related to fear extinction processes, such as harm expectancy or contingency beliefs.

Only very recently has research begun to assess the role of intolerance of uncertainty (IU) in fear extinction (Dunsmoor, Campese, Ceceli, LeDoux, & Phelps, In press; Morriss, Christakou, & van Reekum, 2015). IU is defined as a dispositional tendency that affects how uncertain situations are perceived and interpreted. Individuals with high IU scores tend to find uncertain situations inherently aversive and anxiety provoking. During experienced uncertainty, high IU individuals may be prone to distorted contingency beliefs, where the expectancy of threat may be disproportionate to the expectancy of safety. This may result in the generalization of potential threat to ambiguous, neutral, or even positive cues (Dugas, Buhr, & Ladouceur, 2004). Originally, IU was considered to be specifically related to Generalized Anxiety Disorder (Dugas et al., 2004). However, growing evidence suggests IU may be a transdiagnostic factor across many anxiety and mood disorders (Carleton, Fetzner, Hackl, & McEvoy, 2013; Gentes and Ruscio, 2011, McEvoy and Mahoney, 2012). Furthermore, the development of new disorder-specific IU scales (Thibodeau et al., 2015) highlights that IU may be applicable to specific phobia and Post-Traumatic Stress Disorder (PTSD), which are associated with compromised fear extinction learning.

In the context of fear extinction learning, uncertainty surrounding unannounced learned contingency changes (i.e. CS-US pairings) may initiate generalized expectancy of potential threat in high IU individuals, resulting in fearful responding to both learned threat and safety cues. In a recent neuroimaging study, during early fear extinction learning, we found high IU scores to be associated with equally high skin conductance to learned threat and safety cues, as well as greater activity within the right amygdala to learned safety vs. threat cues, suggesting threat generalization. Furthermore, in late extinction learning, high IU scores were associated with continued fear expression to learned threat vs. safety cues, indexed by larger skin conductance and right amygdala activity (Morriss et al., 2015). Given these recent findings outlined above, it seems pertinent to further examine whether IU proves to be a more sensitive predictor of compromised fear extinction, over general trait anxiety measures such as the STAI. Understanding associations between IU and fear extinction learning could help characterize specific IU-related cognitive biases that disrupt fear extinction processes, such as expectancy of potential threat that may impede the re-establishment of a previously paired CS+ as safe, with implications for targeted treatment, with implications for targeted treatment (Dugas & Robichaud, 2007; Dunsmoor et al., In press; van der Heiden, Muris, & van der Molen, 2012).

Here we used cued fear conditioning to assess the relationship between individual differences in self-reported IU and in psychophysiological correlates of fear extinction learning over time. We measured skin conductance response (SCR) and self-reported uneasiness whilst participants performed the conditioning task. We used an aversive sound as an unconditioned stimulus and visual shapes as conditioned stimuli, as in previous conditioning research (Barrett & Armony, 2009; Büchel, Morris, Dolan, & Friston, 1998; Delgado, Nearing, LeDoux, & Phelps, 2008; Neumann and Waters, 2006, Phelps et al., 2004). We hypothesized that, during fear extinction learning, future threat uncertainty sensitivity would predict generalized fear expression to both learned threat and safety cues, and/or sustained fear expression to learned threat cues (Morriss et al., 2015). Given that fear extinction paradigms are temporally sensitive (Gazendam et al., 2013, LaBar et al., 1998, Milad and Quirk, 2012, Phelps et al., 2004, Sehlmeyer et al., 2011), we expected this effect to be indexed by: (1) Larger responses in high IU individuals to both learned threat and safety cues in *early* fear extinction, across SCR and self-reports, and (2) sustained responses in high IU individuals to learned threat cues vs. safety cues during *late* fear extinction, across SCR and self-reports. Similar to our previous work (Morriss et al., 2015), we tested the specificity of the involvement of IU by comparing it with broader measures of anxiety, such as Spielberger State-Trait Anxiety Inventory, Trait Version (STAIX-2) (Spielberger et al., 1983) and Penn State Worry Questionnaire (PSWQ) (Meyer, Miller, Metzger, & Borkovec, 1990).

## 2 Method

### 2.1 Participants

38 students took part in this study (age range = 18–25 years; 32 females & 6 males). All participants had normal or corrected to normal vision and could only take part if they were in between 18 and 25 years of age. Participants provided written informed consent and received course credit for their participation. Participants were recruited through advertisements and the University of Reading Psychology Panel. The procedure was approved by the University of Reading Ethics Committee.

### 2.2 Procedure

Participants arrived at the laboratory and were informed on the procedures of the experiment. Firstly, participants were taken to the testing booth and given a consent form to sign as an agreement to take part in the study. Secondly, to assess emotional disposition we asked participants to complete a series of questionnaires presented on a computer in the testing booth. Next, physiological sensors were attached to the participants’ non-dominant hand. Participants were simply instructed to: (1) maintain attention to the task by looking and listening to the colored squares and sounds presented, (2) respond to the uneasiness scale that followed each trial (see “Conditioning task” below for details) using the keyboard with their dominant hand and (3) to sit as still as possible. Participants were presented a conditioning task on the computer, whilst electrodermal activity, interbeat interval and ratings were recorded. After the task, subjects were asked to rate the valence and arousal of the sound stimulus using 9-point Likert scales ranging from 1 (Valence: very negative; Arousal: calm) to 9 (Valence: very positive; Arousal: excited). All together, the experiment took approx. 1 h.

### 2.3 Conditioning task

The conditioning task was designed using E-Prime 2.0 software (Psychology Software Tools Ltd, Pittsburgh, PA). Visual stimuli were presented using a screen resolution of 800 × 600 with a 60Hertz refresh rate. Participants sat at approximately 60 cm from the screen. Sound stimuli were presented through headphones.

Visual stimuli were light blue and yellow squares with 183 × 183 pixel dimensions that resulted in a visual angle of 5.78° × 9.73°. The aversive sound stimulus consisted of a fear inducing female scream (sound number 277) from the International Affective Digitized Sound battery (IADS-2) and which has been normatively rated as unpleasant (*M* = 1.63, *SD* = 1.13) and arousing (*M* = 7.79, *SD* = 1.13) (Bradley & Lang, 2007). We used Audacity 2.0.3 software (http://audacity.sourceforge.net/) to shorten the female scream to 1000 ms in length and to amplify the sound by 15 db, resulting in a 90 db (∼5 db) sound. An audiometer was used before testing to standardize the sound volume across participants.

**Fig. 1:**
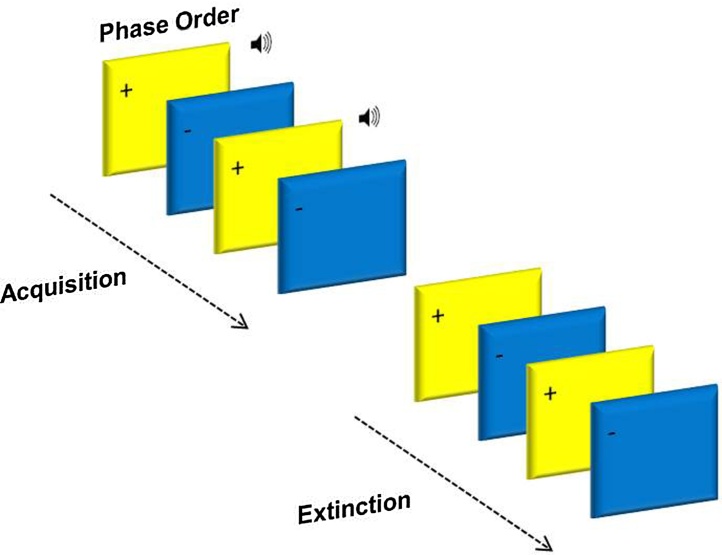
Conditioning task design.

Acquisition and extinction phases were presented in two separate blocks (see Fig. 1). In acquisition, one of the squares (blue or yellow) was paired with the aversive 90 db scream 100% of the time (CS+), whilst the other square (yellow or blue) was presented alone (CS−). In extinction, both stimuli were unpaired (CS+, CS−). The third phase was a partial reacquisition, CS+ squares were paired with the sound 25% of the time, and the CS− remained unpaired (results not reported here).

The acquisition phase consisted of 24 trials (12CS+, 12CS−), the extinction phase 32 trials (16CS+, 16CS−) and the reacquisition 30 trials (16CS+ (4 unpaired)_,_ 14CS−; results not reported here). Experimental trials within the conditioning task were pseudo-randomized into an order, which resulted in no more than three presentations of the same stimulus in a row. Conditioning contingencies were counterbalanced, with half of the participants receiving the US with a blue square and the other half of participants receiving the US with a yellow square.

The presentation times of the task were: 1500 ms square, 1000 ms sound (played 500 ms after the onset of a CS+ square), 3000–6450 ms blank screen, 4000 ms rating scale, and 1000–2500 ms blank screen (see Fig. 1). The uneasiness rating scale asked how ‘uneasy' the participant felt after each stimulus presentation, where the scale was 1 ‘not at all' − 9 ‘extremely'.

### 2.4 Questionnaires

To assess emotional disposition, we presented the following six questionnaires on a computer: Two versions of the Positive and Negative Affect Scales (PANAS-NOW; PANAS-GEN) (Watson, Clark, & Tellegen, 1988), Spielberger State-Trait Anxiety Inventory, Trait Version (STAIX-2) (Spielberger et al., 1983), Penn State Worry Questionnaire (PSWQ) (Meyer et al., 1990), Intolerance of Uncertainty (IU) (Buhr & Dugas, 2002) and the Barratt Impulsiveness Scale (BIS-11) (Patton & Stanford, 1995). We focused on IU because of the intrinsic uncertainty within conditioning paradigms. The IU measure consists of 27 items, example items include “I must get away from all uncertain situations” and “Uncertainty makes me uneasy, anxious, or stressed”. Similar distributions and internal reliability of scores were found for the anxiety measures, IU (*M* = 63.92; *SD* = 19.56; *range* = 31–116; α = 0.94), STAIX-2 (*M* = 44.02; *SD* = 9.33; *range* = 31–65; α = 0.90) and PSWQ (*M* = 51.60; *SD* = 11.56; *range* = 29–71; α = 0.88). Notably, the psychometric properties of the IU scale here match those presented in previous IU validation studies (Buhr and Dugas, 2002, Dugas et al., 2004). We collected the other questionnaires to check for correlational consistency and specificity across anxiety measures, as well as to check for outlying values on IU due to mood or impulsivity.

### 2.5 Rating data scoring

Rating data were reduced for each subject by calculating their average responses for each experimental condition using the E-Data Aid tool in E-Prime (Psychology Software Tools Ltd, Pittsburgh, PA).

### 2.6 Physiological acquisition and scoring

Physiological recordings were obtained using AD Instruments (AD Instruments Ltd, Chalgrove, Oxfordshire) hardware and software. Electrodermal activity was measured with dry MLT116F silver/silver chloride bipolar finger electrodes that were attached to the distal phalanges of the index and middle fingers of the non-dominant hand. A low constant-voltage AC excitation of 22 mV_rms_ at 75 Hz was passed through the electrodes, which were connected to a ML116 GSR Amp, and converted to DC before being digitized and stored. Interbeat Interval (IBI) was measured using a MLT1010 Electric Pulse Transducer, which was connected to the participant’s distal phalange of the ring finger. An ML138 Bio Amp connected to an ML870 PowerLab Unit Model 8/30 amplified the electrodermal and interbeat interval signals, which were digitized through a 16-bit A/D converter at 1000 Hz. IBI signal was used only to identify movement artefacts and was not analyzed. The electrodermal signal was converted from volts to microSiemens using AD Instruments software (AD Instruments Ltd, Chalgrove, Oxfordshire).

Skin conductance responses (SCR) were scored when there was an increase of skin conductance level exceeding 0.03 microSiemens. The amplitude of each response was scored as the difference between the onset and the maximum deflection prior to the signal flattening out or decreasing. SCR onsets and respective peaks were counted if the SCR onset was within 0–7 s following the CS onset.^1^ Trials with no discernible SCRs were scored as zero. The first trial of each experimental phase was excluded, to reduce contamination of averages from the unusually large SCR that typically occurs at the start of a session. SCR amplitudes were square root transformed to reduce skew. Trials with motion artefacts, as identified by distortions in both electrodermal and IBI signals, were discarded from the analysis. 1.3% (26 out of 1904) trials were removed from the analysis due to movement artefacts. SCR magnitudes were calculated from remaining trials by averaging SCR square root transformed values and zeros for each condition. In acquisition, 33% of trials were scored as zero responses and in extinction 53% of trials were scored as zero responses

### 2.7 Learning assessment

To assess whether participants learned the association between the neutral cue and aversive sound, we calculated a conditioned response score for ratings and SCR magnitude in extinction. The conditioned response score was the first 2CS+ trials − the first 2CS− trials, similar to previous work assessing conditioned responses in extinction (Dunsmoor et al., In press; Milad et al., 2009, Phelps et al., 2004). We calculated a conditioned response during the first two trials of extinction because during the acquisition phase, which used a 100% reinforcement schedule, the response would be confounded by the sound presentation. A positive differential response score indicated a larger response for CS+ relative to CS−, indexing a conditioned response. Based on this criterion, only three participants out of the thirty-eight participants were considered non-learners because they did not display a differential response in either ratings or SCR magnitude. However, as removing them did not change the results reported here, we decided to include these three participants for reasons of completeness.

### 2.8 Rating and SCR magnitude analysis

IU-related differences across extinction were assessed by conducting a Condition (CS+, CS−) × Time (Early, Late) × IU repeated measures ANCOVA for the ratings and SCR magnitude, where IU was entered as a continuous mean centered predictor variable. The early part of extinction was defined as the first eight CS+ and eight CS− trials, and the last part of extinction was defined as the last eight CS+ and eight CS− trials. We performed follow-up pairwise comparisons on the estimated marginal means, adjusted for IU. Any interaction with IU was followed up with pairwise comparisons of the means between the conditions for IU estimated at the specific values of + or − 1 SD of mean IU. These data are estimated from the ANCOVA of the entire sample, not unlike performing a simple slopes analysis in a multiple regression analysis. To check for specificity of findings with IU in extinction, we conducted a Condition (CS+_,_ CS−) × IU repeated measures ANCOVA on the ratings and SCR magnitude obtained in the acquisition phase. We did not include both acquisition and extinction phases into one omnibus model because the CS+ is not comparable across phases, given that in the acquisition phase the CS+ is always paired with the US and in the extinction phase the CS+ is always unpaired.

We performed hierarchical regression analyses on the resulting significant SCR magnitude and rating difference scores (CS+ − CS− early; CS+ − CS− late; CS+ early − CS+ late; CS− early − CS− late) for extinction and the anxiety measures to test for IU-specific effects over and above the variance shared with trait anxiety. We entered STAIX-2 and PSWQ in the first step and then IU in the second step.

## 3 Results

### 3.1 Ratings

One participant’s task rating data were missing due to a recording error, leaving rating data for 37 participants. All remaining participants rated the sound stimulus as aversive (M = 2.33, SD = 1.56) and moderately arousing (M = 6.97, SD = 1.48), in accordance with the normative data provided with the IADS-2 set (Bradley & Lang, 2007).

During acquisition participants significantly reported feeling more uneasy for the CS+ vs. CS− trials, *F*(1,35) = 105.993, *p* < 0.001, *η^2^* = 0.75 (see Table 1).

During extinction, participants reported feeling significantly more uneasy to the CS+ vs. CS− trials across extinction, *F*(1,35) = 17.121, *p* < 0.001, *η^2^* = 0.32. In addition, there was a significant interaction of Condition × Time, *F*(1,35) = 6.146, *p* = 0.016, *η^2^* = 0.13, revealing participants’ uneasiness ratings to be higher to the CS+ vs. CS− during the early part of extinction, *p* < 0.001, relative to the late part of extinction, *p* = 0.007 (for descriptive statistics of ratings, see Table 1). Furthermore, participants also reported feeling more uneasy at the start of extinction in general, compared to the end of extinction *F*(1,35) = 36.492, *p* < 0.001, *η^2^* = 0.51.

Contrary to predictions, results revealed no effect of IU for the ratings in any of the experimental phases, *p*’s > 0.3, *F*’s < 0.1,5, max *F* = 1.031.

### 3.2 SCR magnitude

4 subjects were removed from the SCR magnitude analysis due to 1 non-responding, 2 excessive movements, and 1 outlier on SCR magnitude from the early fear extinction CS+ vs. CS− difference score that was +6 SD from the group mean, leaving 34 participants.

As expected, CS+ stimuli elicited larger SCR magnitudes than CS− during acquisition, *F*(1,32) = 118.114, *p* < 0.001, *η^2^* = 0.79 (see, Table 1). There was no interaction between Condition × IU, *F*(1,32) = 0.016, *p* = 0.900, *η^2^* = 0.001.

During extinction, SCR magnitude was on average greater for the CS+ vs. CS−, suggesting participants learned the CS-US contingency, *F*(1,32) = 8.972, *p* = 0.005, *η^2^* = 0.22 (see Table 1). Additionally, SCR magnitude decreased as a function of time for both conditions, *F*(1,32) = 5.667, *p* = 0.023, *η^2^* = 0.15. However, no significant Condition × Time interaction was found, *F*(1,32) = 1.417, *p* = 0.243, *η^2^* = 0.04.

**Fig. 2:**
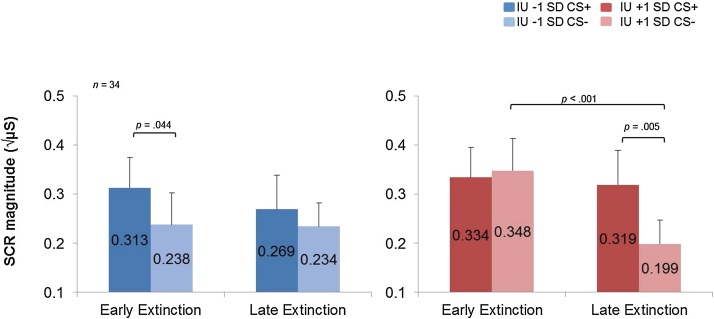
Bar graphs depicting IU estimated at + or − 1 SD of mean IU during early and late fear extinction learning. Low IU scores were associated with significantly greater SCR magnitude responses to CS+ vs. CS− in early extinction, and no differences between stimuli in late extinction, suggesting typical fear expression and extinction respectively. High IU scores were associated with no SCR magnitude discrimination between CS+ and CS− in early extinction, but did show SCR magnitude discrimination between CS+ and CS− in late extinction, as well as a reduction in SCR magnitude to CS− in early vs. late extinction, suggesting threat generalization and compromised safety learning. Square root transformed SCR magnitude (√mS), skin conductance magnitude measured in microSiemens. Standard error bars represent standard error estimated a + or − 1 SD of mean IU.

Taking into account individual differences in IU we found, as predicted, a significant Condition × Time × IU interaction, *F*(1,32) = 4.719, *p* = 0.037, *η^2^* = 0.12, in extinction. Further inspection of follow-up pairwise comparisons for early vs. late extinction at IU ±1 SD from the mean on the regression line showed lower IU (1 SD below the mean) to be associated with significantly greater SCR magnitude in early extinction to the CS+, relative to the CS−, *p* = 0.044, which dissipated over time (late extinction CS+ vs. CS−, *p* = 0.378) (see, Fig. 2). In contrast, higher IU (1 SD above the mean) was associated with no significant differences in early extinction between the CS+ and CS−, *p* = 0.718. In late extinction, higher IU was associated with larger SCR magnitude to the CS+, relative to the CS−, *p* = 0.005 (see Fig. 2). Furthermore, high IU predicted a significant reduction in SCR magnitude to CS− in late extinction, relative to CS− in early extinction, *p* < 0.001. No other significant main effects or interactions were found with IU, *p*’s > 0.1, Max *F* = 1.636.

We conducted hierarchical regression analyses on the effects that were significant in the ANCOVA above. Hierarchical regression analyses of early and late SCR magnitude difference scores in extinction revealed mixed specificity with IU over the STAIX-2 and PSWQ measures. We found no specificity of IU, over STAI and PSWQ measures for the CS+ vs. CS− early and late extinction difference scores (see Table 2). However, we did find specificity for IU, over and above the STAIX-2 and PSWQ measures for CS− early − CS− late extinction difference scores (see Table 2).

## 4 Discussion

In the present study, we show that self-reported IU predicts generalized fear expression to both learned threat and safety cues. These results replicate and extend prior findings from our lab of bodily and neural responding associated with IU and fear extinction (Morriss et al., 2015). These findings suggest that IU-related mechanisms may play a critical role in disrupting fear extinction processes and maintain extinction-resistant fear in anxiety disorders such as specific phobia and PTSD.

Consistent with previous research examining IU and fear extinction (Morriss et al., 2015), low IU was associated with larger SCR magnitude to learned threat cues, relative to safety cues during early extinction, and no differences in SCR magnitude between learned threat and safety cues during late extinction. Expanding previous research on individual differences in trait anxiety (Barrett and Armony, 2009, Gazendam et al., 2013; Indovina, Robbins, Núñez-Elizalde, Dunn, & Bishop, 2011; Sehlmeyer et al., 2011) and IU (Dunsmoor et al., In press; Morriss et al., 2015), we found high IU to be associated with increased SCR magnitude to both learned threat and safety cues during early extinction and larger SCR magnitude to learned threat cues, relative to safety cues in late extinction. Furthermore, high IU was uniquely associated with a reduction in SCR magnitude to learned safety cues from early to late extinction. This latter effect was specific to IU, over STAIX-2 and PSWQ measures. In our previous neuroimaging study, we did not find IU specificity for this effect in physiological indices but we did for right amygdala activity (Morriss et al., 2015). From this, we can speculate that larger SCR magnitude for early safety cues vs. late safety cues in our current study is driven by heightened responsivity in the amygdala. Taken together, these results suggest that IU may play an important role in modulating fear extinction processes such as contingency beliefs and harm expectancy. From these findings, we can speculate that high IU individuals may be prone to biases in the expectancy of potential threat. This may have implications for anxiety disorders that are associated with heightened arousal to learned threat such as specific phobia and PTSD. However, further work is needed to examine how and which IU-related cognitive biases specifically disrupt fear extinction processes.

Contrary to our earlier work involving brain imaging (Morriss et al., 2015), in this study, IU shared variance with STAIX-2 and PSWQ in predicting differential SCR magnitude to learned threat vs. safety cues during fear extinction. The reasons for discrepant findings in specificity between the two studies may be due to: (1) quality of physiological measures inside and outside the scanner, (2) differences in samples sizes, and, (3) IU score ranges, and highlight a further need to study IU in extinction in highly powered experiments.

Self-reported uneasiness ratings were not found to reflect individual differences in IU in our sample. Differences between self-reported and psychophysiological measures of emotion are often reported (Mauss, Levenson, McCarter, Wilhelm, & Gross, 2005), perhaps due to lack of sensitivity of self-report metrics to capture such individual differences. Since we found that IU predicted psychophysiological responding, we think that IU is a more suitable predictor of bodily responses during fear extinction, capturing both unconscious and conscious processing, than moment-to-moment subjective ratings of uneasiness which only capture consciously felt changes in state. However, the lack of relationship between psychophysiological and ratings may also be due to the time between phasic cue events and rating periods, where ratings incorporate an element of recall.

We found no evidence of IU predicting differential psychophysiological responses during fear acquisition for the threat and safety cues. However, we used a 100% reinforcement schedule in the acquisition phase, where the CS+ and US are confounded. Furthermore, the 100% reinforcement schedule is very certain and unambiguous. Therefore, high IU individuals are not generally more aroused to the US and do not generalize fear to CS− cues during acquisition, at least during 100% reinforcement. Further work needs to specifically test whether high IU individuals also show discriminatory deficits during the acquisition of conditioned fear (Dunsmoor et al., In press; Gazendam et al., 2013, Indovina et al., 2011).

Our study has a number of limitations that need to be considered when interpreting the findings presented. Firstly, the study was conducted on a young, predominantly female, student sample, which may limit the generalizability of the results. Secondly, as noted above, we used a 100% reinforcement schedule during fear acquisition. Therefore, we assessed CS-US learning at the start of the extinction phase. Thirdly, in the current study we used a short CS-US interval of 500 ms. Therefore, we could not decouple CS and US omission responses (Bach, Friston, & Dolan, 2013; Spoormaker et al., 2012). Separating CS and US omission responses in future studies may elucidate exactly what aspect of learning (CS vs. US omission responses) is associated with compromised fear extinction in high IU individuals.

In conclusion, individual differences in IU predicted fear expression during extinction. High IU was associated with elevated fear expression to both threat and safety cues during early extinction, and showed continued fear expression to threat cues during late extinction. These findings suggest that high IU individuals are more prone to generalizing learned threat when uncertain, which subsequently compromises fear extinction learning. Importantly, these results highlight an opportunity for further research to examine how individual differences in IU may modulate cognitive biases, particularly that of expectancy bias, in fear and anxiety (Aue & Okon-Singer, 2015). Additionally, these results show promise for the further development of recently implemented focused forms of anxiety disorder treatment, such as intolerance of uncertainty therapy (van der Heiden et al., 2012) and novel experimental models of targeted therapies (Dugas et al., 2004; Dunsmoor et al., In press) in those demonstrating IU-based symptomatology that could specifically help manage uncertainty-based maintenance of learned fear.

## Conflict of interest

The authors declare no conflict of interest.

## Figures and Tables

**Table 1:** Summary of means (SD) for each dependent measure as a function of condition, separately for acquisition and extinction.

Measure	Acquisitio		Extinction		Early Extinction		Late Extinction	
	CS+	CS−	CS+	CS−	CS+	CS−	CS+	CS−
Square root transformed SCR magnitude (√μS)	0.79 (0.33)^b^	0.32 (0.25)^a^	0.31 (0.24)^d^	0.25 (0.22)^c^	0.32 (0.25)	0.29 (0.26)	0.29 (0.28)	0.22 (0.20)

Uneasiness rating (1–9)	6.14 (1.73)^b^	3.10 (1.73)^a^	2.70 (1.25)^d^	2.14 (1.09)^c^	3.12 (1.28)^f^	2.41 (1.30)^e^	2.28 (1.35)^h^	1.86 (.98)^g^

Note: SCR magnitude (√μS), square root transformed skin conductance magnitude measured in microSiemens. Superscripts indicate significant (*p* < 0.05) condition difference from: ^a^ Acquisition CS+, ^b^ Acquisition CS−, ^c^ Extinction CS+, ^d^ Extinction CS−, ^e^ Early Extinction CS+, ^f^ Early Extinction CS−, ^g^ Late Extinction CS+, ^h^ Late Extinction CS−.

**Table 2:** Summary of hierarchical regression analyses for anxiety measures predicting extinction difference scores.

Predictors	CS+ − CS− Early Extinction						CS+ − CS− Late Extinction						CS− Early Extinction − CS− Late Extinction					
	*B*	*SE B*	β	*R^2^*	*F*	Δ *R^2^*	*B*	*SE B*	β	*R^2^*	*F*	Δ *R^2^*	*B*	*SE B*	β	*R^2^*	*F*	Δ *R^2^*
Step 1				0.074	1.232	0.074				0.061	1.008	0.061				0.089	1.7	0.089
STAI	0.003	0.003	−0.39				0.006	0.004	0.322				0.007	0.004	0.439			
PSWQ	−0.006	0.004	0.258				−0.002	0.004	−0.125				−0.005	0.003	−0.377			

Step 2				0.12	1.571	0.046				0.073	0.386	0.012				0.298	10.124	0.209*
STAI	−0.001	0.006	−0.086				0.003	0.006	0.167				−0.002	0.005	−0.123			
PSWQ	0.003	0.003	0.255				−0.002	0.004	−0.124				−0.006	0.003	−0.45			
IU	−0.003	0.002	−0.371				0.002	0.003	0.188				0.006	0.002	0.77			

*Note:* * *p* <  0.01.

## Footnotes

1 The SCR magnitude results of the study do not change when we include only those SCR onsets within 0–4.5 s after CS onset. The main effect of Condition for SCR magnitude with 7 s SCR onsets, *F*(1,32) = 8.972, *p* = 0.005 vs. SCR magnitude with 4.5 s SCR onsets, *F*(1,32) = 11.593, *p* = 0.002. Similarly, Condition × Time × IU interaction for SCR magnitude with 7 s SCR onsets, *F*(1,32) = 4.719, *p* =0.037 vs. SCR magnitude with 4.5 s SCR onsets, *F*(1,32) = 4.666, *p* = 0.038. The IU-related findings during fear extinction were not driven by the late SCR onset times. We conducted a 2 (Condition: CS+, CS−) × 7 (SCR Onset Time: 0-1,1-2,2-3,3-4,4-5,5-6,6-7) repeated measures ANCOVA with IU entered as a covariate. There was a significant main effect of Condition, *F*(1,32) = 5.841, *p* = 0.022, and of SCR Onset Time, *F*(6,192) = 2.946, *p* = 0.009. These effects were driven by there being a higher number of responses overall to the CS+ vs. CS−, *p* = 0.022. A higher number of responses were observed with onsets between 1 and 2 and 2–3 s, compared to 4–5, 5–6, and 6–7 s, *p*’s < 0.05. There were no significant interactions between Condition × Onset Time, Condition × IU, Onset Time × IU, or Condition × Onset Time × IU, max *F* = 1.291, *p*’s > 0.2.
